# Nest concealment is associated with reproductive traits across sympatric bird species

**DOI:** 10.1002/ece3.8117

**Published:** 2021-09-17

**Authors:** Jinlong Liu, Han Yan, Guopan Li, Shaobin Li

**Affiliations:** ^1^ Engineering Research Center of Ecology and Agricultural Use of Wetland, Ministry of Education College of Life Sciences, Yangtze University Jingzhou China; ^2^ MOE Key Laboratory of Biodiversity and Ecology Engineering Beijing Normal University Beijing China

**Keywords:** breeding biology, life history, nest‐site selection, phylogenetically informed analysis, Tibet Plateau

## Abstract

Nest‐site characteristics are thought to play an important role in reproductive performance in birds (e.g., influencing reproductive success and predation risk). Nest‐site characteristics such as concealment may be particularly critical at high elevation where nests are exposed to challenging environmental conditions. In this study, we conducted both conventional and phylogenetically controlled analyses to investigate whether nest concealment affected several reproductive traits across 21 sympatric bird species living on Tibet Plateau (3,400 m altitude). Qualitatively equivalent results were reached in analyses, regardless of phylogenetic controls. We found that clutch size, incubation period, nestling period, and nest success were strongly and positively associated with nest concealment across species. Our study addressed such a high‐elevation bird community that is lacking in the previous studies. This study adds to theory that while there are a few exceptions, overall evidence supports a positive effect of nest concealment on reproductive performance across coexisting alpine species.

## Graphical abstract

Phylogenetic comparative analyses and conventional analyses suggest that nest concealment positively affects reproductive traits across sympatric species at high altitude. This study adds to the evidence that there is a positive effect of nest concealment on reproductive performance across species.

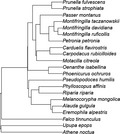

## 1 INTRODUCTION

Nest predation is the primary cause of nest failure across a wide diversity of bird species and habitats, so nest concealment (including nest characteristics such as location and appearance) can be important for birds to enhance their reproductive performance (Martin, 1993; Ricklefs, 1969; Roff, 2002; Signorell et al., 2010). Reproductive performance can be subdivided into several different components, including clutch size, growth and development rate, and nest survival, all of which are important reproductive traits in birds (Roff, 2002). A well‐concealed site can minimize the transmission of auditory, visual, and olfactory cues from the nest to potential predators (Martin, 1993), and numerous studies have attempted to identify nest‐site characteristics that may affect these reproductive traits (reviewed in Burhans & Thompson, 1998; Martin, 1992). Some studies found positive effects of nest concealment on reproductive performance (Grendelmeier et al., 2015; Martin, 1992; Martin et al., 2000; Remeš, 2005; Weidinger, 2002), whereas others found no effects (Burhans et al., 2002; Howlett & Stutchbury, 1997; Hu et al., 2017; Li, Qin, et al., 2018; Smith et al., 2018). This difference can be attributed to factors such as predator type (olfactory vs. visual). Birds facing olfactory predators (e.g., snakes or mammals) often have no relation between nest concealment and nest success when their nests are accessible (Conover et al., 2010; Oswald et al., 2020).

A number of studies have conducted interspecific analyses to test the effect of nest concealment on life‐history traits (e.g., Martin, 1995; Martin et al., 2017; Martin & Li, 1992; Söderström et al., 1998; Weidinger, 2004), but most of these earlier analyses were performed without controlling for the phylogenetic relationships between the species concerned. This may lead to illusory relationships between nest‐site characteristics and reproductive traits because of the lack of statistical independence among species (Felsenstein, 1985; Harvey & Pagel, 1991). Therefore, interspecific analyses that control for phylogeny are required to reduce potential biases in predicting a relationship among variables (Freckleton et al., 2002). Borgmann and Conway (2015) conducted phylogenetically controlled analyses regarding nest concealment on reproductive traits across species of different regions but yielded equivocal results. Their study selected only open‐cup nest birds and used foliage features as indices of nest concealment. In addition, the methodology of nest concealment measurement was quite different across species (Borgmann & Conway, 2015).

For avian species, building the nest in a location that is difficult for predators to access or locate is an effective way to minimize nest predation (Martin, 1993; Roff, 2002; Weidinger, 2004). Therefore, in addition to foliage features and the nest types that offer differing levels of nest concealment (Martin, 1995; Watters et al., 2002), other nest‐site characteristics should be taken into account for interspecific analyses, such as nest location in the landscape (e.g., on ground, in bush, or on cliff) and tunnel length for cavity nests (mostly burrows dug into the ground or cliff). For example, the extent of nest concealment may differ between open‐cup nests on the ground and in thorny shrubs above the ground (Campos et al., 2011; Martin, 1987), and among cavity nests opening on flat ground and on to cliff banks (Li & Lu, 2012b; Li, Peng, et al., 2015).

The majority of previous studies suggest that species with better concealed nest have larger clutch sizes and greater nest success because they suffer lower predation rates (Li & Lu, 2012a, 2012b; Martin & Li, 1992). Larger clutches in better concealed nests could evolve as a covariate of reduced nestling growth rates permitted by lower predation rates (Lack, 1968). In this paper, we collected data on several reproductive traits and the nest characteristics of 21 sympatric bird species in a high‐elevation habitat on the Tibetan Plateau.

High‐elevation communities are understudied in terms of nest characteristics and predation risk. Species living sympatrically likely experiences a similar biotic and abiotic environment, including climate and predation risk; an interspecific analysis of sympatric species thus provides an opportunity to examine biological traits while controlling for the confounding effects of biotic/abiotic factors. The habitat at high elevation tends to be more homogenous (Li, Qin, et al., 2018; Wang & Lu, 2018), so there are fewer options for nest placement than in a forest community and open‐cup nests in particular are more exposed. The challenging climate of high‐elevation habitats is also likely a factor in driving the evolution of nest concealment (e.g., constraints on thermoregulation and parental care; Ke and Lu, 2009). Greater exposure could increase predation risk, and thus, there should be selective pressures for birds to place their nests in more covered or inaccessible sites.

Previous interspecific analyses on the effect of nest concealment were mostly based on species of different regions or without controlling for phylogeny. Species living sympatrically can experience similar biotic/abiotic conditions, and a number of confound climate factors can be excluded. Here, we conducted both phylogenetically controlled and conventional analyses to assess the relationship between nest concealment and reproductive traits in sympatric species near the upper limit of their breeding distribution which were not included in former studies. The main objectives of this study were to test the relationship between nest concealment and clutch size, duration of the incubation period, duration of the nestling period, and nest success. We predicted that species with well‐concealed nests would have greater nest success, allow larger clutch sizes but lower growth rate (e.g., longer incubation and nestling periods).

## 2 METHODS AND STUDY SITE

Data were collected either from fieldwork during the current study or from published studies (details in Table 1) conducted in the same area. The collated data mainly included four reproductive traits (clutch size, incubation period, nestling period, and nest success; details on their definitions can be found in Methods below) and several nest‐site attributes (details also in Methods below).

**TABLE 1:** Several reproductive traits of 21 bird species in sympatric area

Species	Clutch size (eggs)	Incubation period (days)	Nestling period (days)	Nest success (proportion)	Nest type	Nest‐site location	Burrow length	No. of nests located	Data quality	Body mass (grams)	References
Tibetan Ground Tit *Pseudopodoces humilis*	6.8	14.1	25	0.910	4	3	2	187	3	39.1	Li (2013), Li, Li, et al. (2015)
Horned Lark *Eremophila alpestris*	2.5	12.4	9.9	0.315	1	1	0	73	3	33.2	Li, Cheng, et al. (2015)
Oriental Skylark *Alauda gulgula*	3.3	12.4	9.7	0.413	1	1	0	46	3	32.5	Li, Peng, et al. (2015)
Mongolian Lark *Melanocorypha mongolica*	3.3	13	10	0.500	1	1	0	4	1	55	Current study
Sand Martin *Riparia riparia*	4.9	14.7	23.3	0.895	4	3	2	19	2	13	Li et al. (2016)
Alpine Leaf‐warbler *Phylloscopus occisinensis*	4.8	13.6	15.3	0.455	2	2	0	11	1	7.3	Current study
Black Redstart *Phoenicurus ochruros*	4.8	13.3	17.25	0.741	3	3	1	27	2	16	Current study
Isabelline Wheatear *Oenanthe isabellina*	5.1	13.5	18.5	0.810	3	1	2	31	3	30	Li and Lu (2012b)
White‐rumped Snowfinch *Montifringilla taczanowskii*	4.6	13.3	21.3	0.833	3	1	2	6	1	40	Current study
Rufous‐necked Snowfinch *Montifringilla ruficollis*	4.2	14	20.5	0.625	3	1	2	8	1	30	Current study
Small Snowfinch *Montifringilla davidiana*	5.8	11.7	19.9	0.860	3	1	2	29	3	21	Li et al. (2013)
Eurasian Tree Sparrow *Passer montanus*	4.2	13.2	15.6	0.722	3	3	1	36	3	21	Current study
Rock Sparrow *Petronia petronia*	5.1	12.7	19.9	0.890	3	3	2	35	3	30	Li and Lu (2012a)
Brown Accentor *Prunella fulvescens*	3.4	12.9	13.9	0.459	1	2	0	37	3	18	Huang et al. (2020)
Rufous‐breasted Accentor *Prunella strophiata*	3.25	13	13.4	0.308	1	2	0	13	2	19.6	Current study
Citrine Wagtail *Motacilla citreola*	4.3	12.2	11.33	0.273	1	1	0	11	2	21.5	Current study
Twite *Carduelis flavirostris*	4.5	12.3	14.3	0.487	1	2	0	47	3	18.5	Current study
Streaked Rosefinch *Carpodacus rubicilloides*	3.5	14.3	14.5	0.462	1	2	0	13	2	41	Current study
Little Owl *Athene noctua*	3	25	27	1.000	3	3	1	4	1	170	Current study
Eurasian Hoopoe *Upupa epops*	5.3	17	28	0.500	3	3	1	4	1	67	Current study
Common Kestrel *Falco tinnunculus*	4.7	27	32.5	1.000	3	3	1	3	1	180	Current study

Note

Nest type: “1” for open‐cup nest, “2” for domed nests (enclosed nests), “3” for cavity nests of nonexcavating birds, and 4 for cavity nests of excavating birds; Nest‐site location: Ground nests were scored as “1,” nests in bushes aboveground as “2,” and nests on steep slopes or banks as “3”; Burrow length: It was scored as “0” for open‐cup nests and enclosed nests, “1” for cavity nests with burrow length of ≤50 cm, and “2” for cavity nests with burrow length >50 cm.

### 2.1 Study site

Fieldwork for novel data was carried out during the 2009–2018 breeding seasons at Tianjun Prairie on the northeast of Tibet Plateau (37°17′N, 99°06′E, 3,400 masl). The study area (ca 600 ha) was mainly public grassland, dominated by alpine steppe meadow, mainly used for livestock grazing. Two shallow streams which originated from the southern mountains run through the grassland. The streams usually dry up until the rainy seasons come from June to July. Mean annual temperature of this area was −0.5 ± 0.7 (range −1.5–0.9)°C and the total annual rainfall 305 ± 64 (range 176–418) mm^3^ (data from 1989 to 2010 from a local weather station). More than 30 bird species (*n* = 7 orders) breed in this area. Mammals (e.g., Siberian Weasel *M. sibirica* and Wild Cat *Felis silvestris*) are predominant predators in our study site (Li, Peng, et al., 2015; Li S's unpublished data). Details on the study site are available in Li, Shi, et al. (2018).

### 2.2 Data collection

We searched the study area for open‐cup and cavity nests from May to August. Nests were located by flushing the incubating individuals, following adults with nest material or following them to a nest during the incubation or nestling periods. When a nest was located, we recorded the date and nest status (presence of parents, eggs, nestlings) with nests then checked every 2–4 days. Open‐cup nests were checked directly while cavity nests were checked either by a pole‐mounted miniature camera or through a hole dug at the side of the nest chamber (previous studies showed no obvious adverse effects from these methods; Li, Shi, et al., 2018). For cavity nests, we recorded the entrance location (on ground or on cliff bank) and measured the burrow length from entrance to the burrow chamber with a measuring tape to 0.1 cm. When a nest was near hatching or fledging, we increased the frequency of checks to every 1–2 days. Nests producing at least one fledgling (still not fully grown, but fully leaving the nest) were considered successful. Nests were assumed to have failed when the nest, eggs, or nestlings disappeared when the length of time since nest initiation suggested they were too young to have fledged.

For each species, we scored three nest‐site attributes. (a) Nest type: as “1” for open‐cup nest, “2” for domed nests (enclosed nests), “3” for cavity nests of nonexcavating birds, and 4 for cavity nests of excavating birds, as predation risk was found to decrease from open‐cup nesters to cavity nesters and from secondary cavity nesters to primary cavity nesters due to nest concealment (Martin & Li, 1992). (b) Nest‐site location: Ground nests were scored as “1,” nests in bushes aboveground as “2,” and nests on steep slopes or banks as “3”; ground nests are assumed to be under higher predation risk than nests aboveground or on cliffs when mammal predators occurred more frequently than avian predators (Söderström et al., 1998; Wilcove, 1985). Previous studies show that mammals are dominant predators in our study site (Li, Peng, et al., 2015; Li's unpublished data). (c) Burrow length: It was scored as “0” for open‐cup nests and enclosed nests, “1” for cavity nests with burrow length of ≤50 cm, and “2” for cavity nests with burrow length of more than 50 cm. We extracted the first component as nest concealment for each species by Phylogenetic Principal Component Analysis (PPCA) for each nest from the three nest‐site attributes, because the first component accounted for 74.6% of the total variance.

We also compiled the data on nest‐site attributes and reproductive traits (clutch size, incubation period defined as the period from the start of incubation to the first egg hatched, nestling period as the period from the first egg hatched to the last young fledged, and nest success) mentioned above from published studies from the same study area. More information on the definitions of the reproductive traits can be found in Li and Lu (2012a, 2012b). All these data were collated from the literature published by our group as we have studied in this area since 2008. A total of 21 species with complete data were collected in the full dataset. Among these sympatric species, datasets of eight species were collected from published studies by our group and datasets of 13 species were from fieldwork (this study; Table 1).

### 2.3 Statistical analyses

Before analysis, we assigned each study a qualitative rank score of data quality from weak (1), medium (2) to strong (3) based on overall impression of data with respect to sample size (roughly *n* ≤ 10 as weak and *n* ≥ 30 as strong) and the details of behavioral observation conducted following Green et al. (2016).

We present and compare the results of both phylogenetically controlled and conventional analyses in line with recommendations from the literature (Oswald et al., 2020; Schluter, 2000; Swanson & Bozinovic, 2011). We first used conventional analysis, fitting general linear models to response variables (e.g., clutch size, incubation period, nestling period, and nest success) as predicted by nest concealment, with body mass as a potential confounding effect. Body mass is either collected from the references in Table 1 or collected from Dunning (2008). Data from these species may be nonindependent for the purposes of statistical analysis due to their common phylogenetic history (Felsenstein, 1985; Harvey & Pagel, 1991). Therefore, we further investigated the effect of nest concealment on reproductive performances across species using phylogenetic generalized least squares (PGLS) models that controlled for phylogeny. All these PGLS analyses were performed with data quality as a weight variable, despite the fact that comparison between these results and those obtained from analyses that omitted the data quality revealed qualitative equivalent results in both cases (all models: *t* > 2.256, *p* < .037).

**FIGURE 1:**
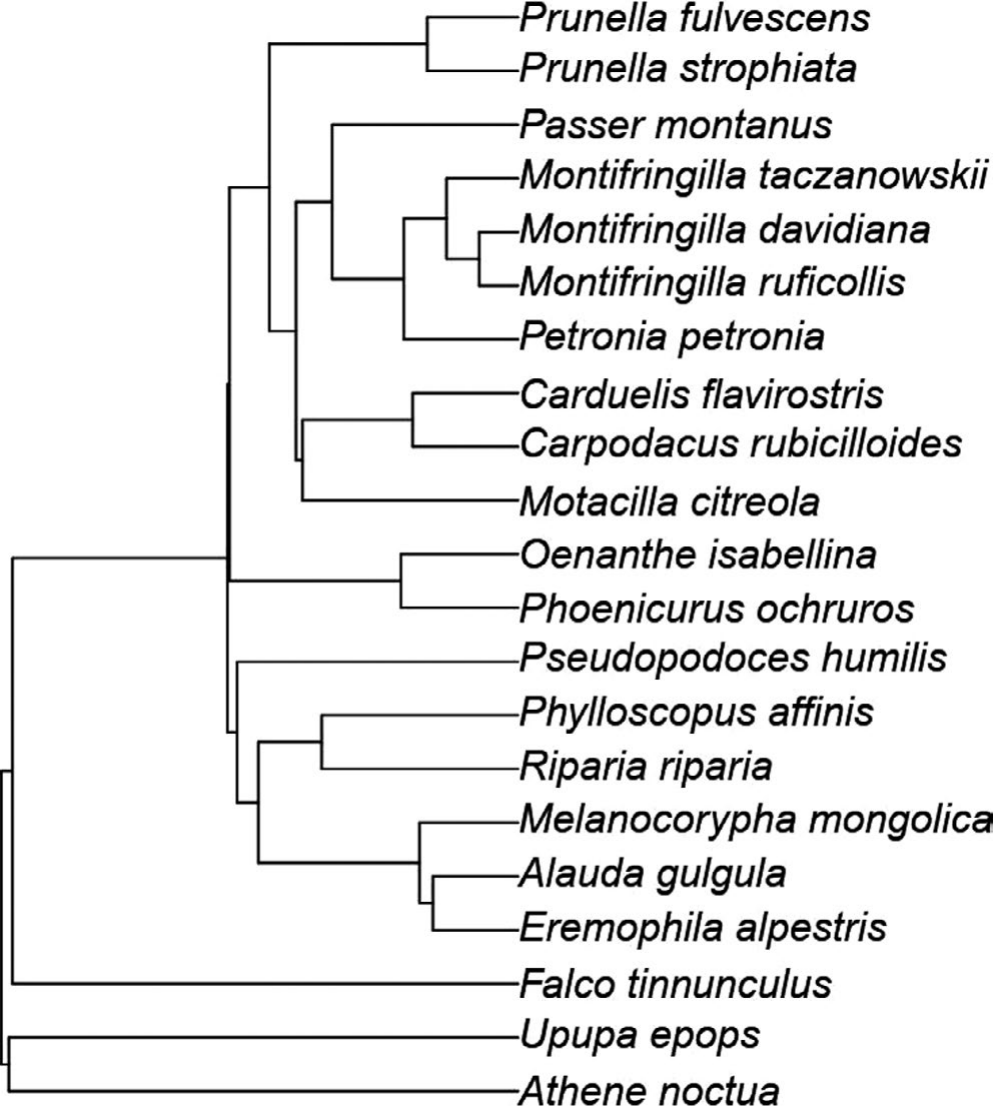
The maximum clade credibility tree (consensus tree) built with the 100 trees from the BirdTree Project for 21 avian species in sympatric area

We downloaded 100 fully resolved trees from BirdTree project (birdtree.org; Jetz et al., 2014) using the Hackett et al. (2008) backbone. One species (Alpine Leaf‐warbler *Phylloscopus occisinensis*) was not included in the bird tree, so we used the phylogenetic position of its closest related species Tickell's leaf warbler (*P. affinis*) instead (Martens et al., 2008). With the 100 trees, we built the maximum clade credibility tree (consensus tree; Figure 1) using R package *phangorn* (Schliep, 2011). PPCA was conducted in R package *phytools* (Revell, 2012). All phylogenic analyses were performed across this summary tree. PGLS models were constructed using the R package *ape* (Paradis et al., 2004).

We applied a maximum‐likelihood estimation of Pagel's *λ* for phylogenetic dependence. Phylogenetic signal (measured as Pagel's *λ*) was tested using restricted maximum likelihood against a value of 0 (the evolution of a trait is independent of phylogeny) (Pagel, 1997). Phylogenetic signal was considered to be present if *λ* differed significantly from 0 (Revell, 2010). The estimated coefficients from the PGLS models reflect the relationship between variables. If the phylogenetic signal is absent or weak, results from both analyses are acceptable; otherwise, phylogenetically informed analyses would be better supported (Freckleton et al., 2002). All statistical analyses were performed with R ver. 4.0.2 (R Core Team, 2018). We report mean ± *SE* and two‐tailed probabilities with .05 significance threshold throughout the paper.

## 3 RESULTS

The mean clutch size and nest success across species were 4.39 ± 1.05 (range 2.5–6.8) eggs and 0.64 ± 0.24 (range 0.27–1.00), respectively. Among the 21 species, twelve were cavity‐nesting birds while the remainder were either open‐cup nesting birds (*n* = 8) or dome‐nesting birds (*n* = 1). For these sympatric species, eight are ground nesters (*n* = 3 on the ground; *n* = 8 in burrows), five placed their nests above the ground in bush, and the others (*n* = 8) built their nests on bank or cliff.

PGLS models that examined the relationship between nest concealment and four reproductive traits (Table 2) produced *λ* values which were significantly different from 0 for two reproductive traits (nestling period and nest success: *λ* > 0.820, *χ* ^2^ > 5.296, *p* < .021). This implies a strong phylogenetic signal for these two correlations (Table 2). However, the λ value between nest concealment and clutch size and incubation period was not significantly different from 0 (Table 2), indicating a weak phylogenetic signal for these traits.

**TABLE 2:** Significance of phylogenetic signal *λ* estimated by restricted maximum likelihood in PGLS models for nest concealment in relation to clutch size, incubation period, nestling period, or nest success when controlling for body mass (*λ* was tested against a value of 0)

Relationship between variables	Null model (*λ* = 0)		
	*λ*	*χ* ^2^	*p*
Clutch size ~Nest concealment	0.637	1.948	.113
Incubation period ~Nest concealment	−0.110	0.037	.847
Nestling period ~Nest concealment	1.065	7.772	.**005**
Nest success ~Nest concealment	0.820	5.296	.**021**

Bold values mean significant effects.

**FIGURE 2:**
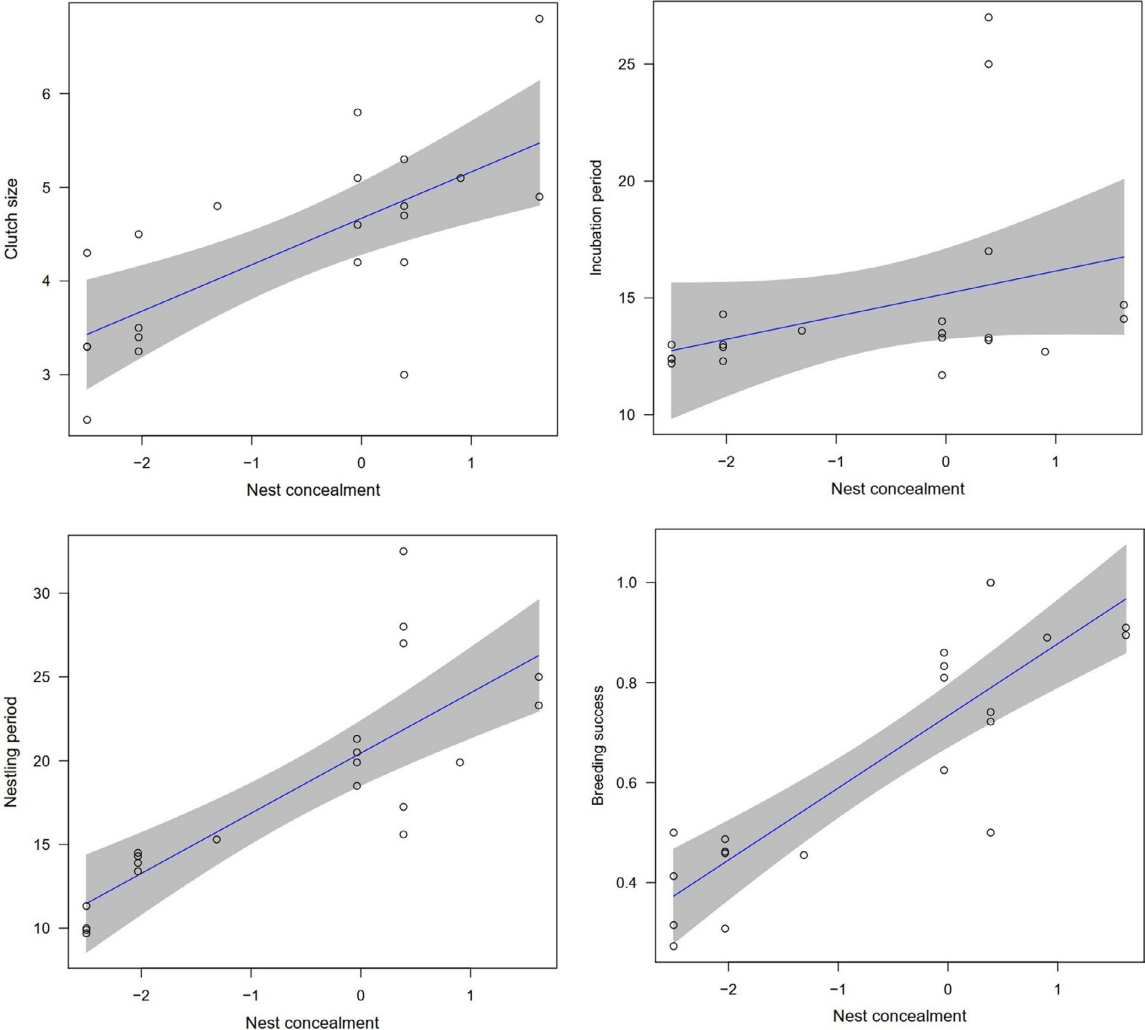
Effect of nest concealment on clutch size (upper left), incubation period (upper right), nestling period (left bottom), and breeding success (right bottom) across 21 sympatric bird species. Data points represent individual species values, regression lines include 95% confidence interval, and model fit is PGLS‐informed GLM

Conventional analyses (general linear models) without phylogenetic controls revealed that nest concealment strongly correlated with each reproductive trait when controlling for body mass and including data quality as a weight variable (all models: *t* > 2.379, *p* < .029; Table 3). Similar to the conventional analysis, phylogenetically informed models yielded qualitatively equivalent results for all these correlations (all models: *t* > 2.461, *p* < .024; Table 3). Across 21 species, clutch size was significantly positively correlated with nest concealment, and the length of incubation and nestling period increased significantly with nest concealment (Table 3, Figure 2). There was also a significantly positive correlation between nest success and nest concealment (Table 3).

**TABLE 3:** Results of the general linear models and PGLS models to determine whether variation in nest concealment affect clutch size, incubation period, nestling period, or breeding success when controlling for body mass across 21 coexisting species

Relationship	General linear models			PGLS models		
	*β* ± *SE*	*t*	*p*	*β* ± *SE*	*t*	*p*
Clutch size ~Nest concealment	0.505 ± 0.121	4.185	**<.001**	0.411 ± 0.116	3.536	.**002**
Incubation period ~Nest concealment	0.490 ± 0.206	2.379	.**029**	0.500 ± 0.203	2.461	.**024**
Nestling period ~Nest concealment	3.461 ± 0.443	7.818	**<.001**	2.866 ± 0.335	8.059	**<.001**
Nest success ~Nest concealment	0.122 ± 0.021	5.759	**<.001**	0.133 ± 0.019	7.060	**<.001**

Note

Body mass as a covariate was positive and significant (*p* < .01) in all analyses regardless of phylogenetic controls.

Bold values mean significant effects.

## 4 DISCUSSION

In this study, we investigated the relationship between nest concealment and four reproductive traits across sympatric species using both phylogenetically informed and conventional analyses. Qualitatively equivalent results were yielded by both approaches: All the reproductive traits (clutch size, incubation period, nestling period, and nest success) were strongly and positively correlated with nest concealment across 21 bird species. This supported our prediction that species with well‐concealed nests would lead to larger clutch sizes but longer incubation and nestling periods, and achieve greater nest success. These findings (the correlation between nest concealment and reproductive traits) are similar to the results from some previous phylogenetic meta‐analyses (Borgmann & Conway, 2015) and nonphylogenetic analyses (Grendelmeier et al., 2015; Martin & Li, 1992; Weidinger, 2002). Our study addressed the relationship between nest concealment and reproductive traits in such a high‐elevation bird community (3,400 asl) that is lacking in the previous literature. Therefore, this study may advance the field and stimulate further study in harsher environment of high‐elevation habitat.

Life‐history theory predicts that clutch size should increase with greater nest success across species (Lack, 1968; Martin & Li, 1992). Reduced nest success is primarily caused by nest predation in bird species, and the general interspecific association of larger clutch size with reduced nest loss is widely accepted (Martin, 1993; Roff, 2002). Our study provides further evidence that increased nest concealment is associated with larger clutch size and greater nest success (Figure 2, Table 3; Martin & Li, 1992). Therefore, in our sample of species, it may be that better concealed nests had lower rates of predation, allowing them to produce larger clutches to increase their future fitness during long evolutionary history.

We also detected a positive and significant effect of nest concealment on incubation period when controlling for body mass. Shorter incubation periods are often found in species that face higher levels of nest predation risk (Li & Lu, 2012b; Martin, 2002; Ricklefs, 1993), so birds may have longer incubation periods when predation risk is lower. Predation risk increases with time in the nest, so if predation risk is lower overall, development can be longer with minimal cost. The selective pressure for a shorter development period is relaxed. This suggests that longer incubation period can be a by‐product of high nest success from better concealed nests. There is also evidence that slower development is adaptive in stochastic environments where severe weather may disrupt resource availability (Arendt, 1997; de Zwaan et al., 2019). Therefore, nest‐site selection that allows for reduced predation risk and longer development times can reflect a balance between both weather and predation risk constraints.

Our results revealed a positive relationship between nest concealment and length of the nestling period (Table 3, Figure 2). Longer nestling periods are usually associated with greater nest success in bird species (Li & Lu, 2012; Martin & Li, 1992) because of weaker selection for rapid nestling growth or development as mentioned in the previous paragraph. In this study, compared with less concealed nests (e.g., open nests on ground), better concealed locations (e.g., cavity nests on cliff) could be more inaccessible for predators. Thus, nestlings in better concealed nests are safer and can have a longer nestling period, which subsequently contribute to better developed fledglings (e.g., well‐developed immune systems) and increased probability of future survivals (Breitwisch, 1989; Roff, 2002). By contrast, reduced fledgling quality from faster development can lead to lower survivorship in some species (Greño et al., 2008; Magrath, 1991; Thompson & Flux, 1991).

As we predicted, better concealed nests are associated with larger clutch sizes, longer incubation and nestling periods, and achieve greater nest success across 21 coexisting species at high elevation. High elevation means adverse environment conditions (e.g., cold weather, strong wind, and thin oxygen) and more homogenous habitat with less option for birds to place their nests. Under these conditions, nest concealment should be more important and thus significantly affect their reproductive traits. To our knowledge, few previous studies have tested the association among nest concealment and reproductive traits at such a high elevation (more than 3,400 masl). This study adds to the evidence that there is a positive effect of nest concealment on reproductive performance at high elevation. However, these analyses should benefit from more species with larger samples. The challenging climate and low breeding density of avian communities at high‐elevation habitats may make it more difficult to collect large number of species with large samples, when compared with studies at low elevations. Longer‐term fieldwork (more species with larger samples) and well‐designed experiments would be particularly helpful to explain the relationship between nest concealment and breeding performance at high elevation in further studies.

## CONFLICT OF INTEREST

The authors declare that they have no competing interests.

## AUTHOR CONTRIBUTION

**Jinlong Liu:** Conceptualization (equal); Writing‐original draft (equal). **Han Yan:** Conceptualization (equal); Formal analysis (equal); Writing‐review & editing (equal). **Guopan Li:** Data curation (equal); Formal analysis (equal). **Shaobin Li:** Conceptualization (lead); Data curation (equal); Supervision (lead); Writing‐original draft (equal).

## Data Availability

All data are included in main text. No other data are needed to be deposited elsewhere.
